# Nutritional related cardiovascular risk factors in patients with coronary artery disease in IRAN: A case-control study

**DOI:** 10.1186/1475-2891-9-70

**Published:** 2010-12-26

**Authors:** Reza Amani, Mohammad Noorizadeh, Samira Rahmanian, Naser Afzali, Mohammad H Haghighizadeh

**Affiliations:** 1 Department of Nutrition, Faculty of Paramedicine, Diabetes Research Centre, Jondi-Shapour University, Ahvaz, Iran; 2 Department of Cardiology, Golestan Medical Centre, Jondi-Shapour University, Ahvaz, Iran; 3 Department of Statistics, Faculty of Public Health, Jondi-Shapour University, Ahvaz, Iran

## Abstract

**Background and aims:**

There are limited findings available on coronary artery disease (CAD) risk factors and nutritional pattern of CAD patients in Iran. The purpose of this study was to compare nutritional-related risk factors of CAD patients with that of matched controls.

**Methods:**

In a case-control design, dietary patterns and CAD risk factors of 108 documented patients (determined by cardiac catheterization showing greater than 70% stenosis or established myocardial infarction) whom were admitted to coronary care units (CCU) of Ahvaz teaching hospitals were compared with that of 108 gender- and age-matched subjects of normal cardiac catheterization (lesser than 40% stenosis). Measured variables consisted of blood lipid profile, smoking habits, dietary patterns, anthropometric indices and blood pressure levels.

**Results:**

Almost all patients had hypertriglyceridemia and high LDL-C levels. Odds ratios (CI 95%) for consuming fish, tea, vegetable oils were 0.55(0.31-0.91), 0.3(0.15-0.65), 0.23(0.13-0.42), respectively. However, consumption of hydrogenated fats, and full-fat yoghurt was associated with higher CAD risk (OR = 2.12(1.23-3.64) and 2.35(1.32-4.18), respectively. Patients' serum lipid profiles, sugar concentrations, and blood pressure levels were significantly higher than defined cut-off points of the known risk factors. Considerable numbers of the control group also showed high levels of the known risk factors.

**Conclusions:**

Consumption of fish, tea and vegetable oils shown to have protective effect on CAD while full fat yoghurt and hydrogenated fats increase the risk of CAD. Moreover, CAD patients obviously have higher blood lipids and sugar concentrations, blood pressure, body fat percent and BMI levels compared with their matched counterparts. We need to define specific local cut-off points with more practical criteria to detect CAD patients.

## Introduction

Coronary artery disease (CAD) is one of the leading causes of death universally and one of the most common chronic illnesses in the developing world [1,2]. Among the conventional cardiovascular risk factors, diet poses a particular challenge for research. Total calorie intake per capita increases as countries develop. With regard to CAD, a key element of dietary change is an increase in intake of saturated animal fats and hydrogenated vegetable fats, which contain atherogenic trans fatty acids, along with a decrease in intake of plant-based foods and an increase in simple carbohydrates. Fat contributes less than 20% of calories in rural China and India, less than 30% in Japan, and well above 30% in the United States. Caloric contributions from fat appear to be falling in the high-income countries [1,2] Indeed, many of the current dietary guidelines for the health of the general population aim to prevent cardiovascular disease. These recommendations emerge from considerable evidence for nutritional influences on cardiovascular disease risk and a smaller but compelling number of studies indicating that certain dietary modifications can reduce the risk. Cross-cultural comparisons, case-control and prospective observational studies have identified a relationship between diet, blood pressure and lipids levels [3-6], but there is still considerable scientific uncertainty about the relationship between specific dietary components and cardiovascular risk especially in Asian populations. Although most of the information about nutritional risk factors and cardiovascular disease derives from studies in the developed world, the situation is rapidly evolving toward epidemic proportions in the developing world where rapid changes have been made in people's lifestyle patterns during recent years [7,8].

One of the widespread criteria that has been used as an estimate of body fat is body mass index (BMI) which is related to risk of disease. Lower BMI thresholds for overweight and obesity have been proposed for the Asia-Pacific region since this population appears to be at-risk at lower body weights for glucose and lipid abnormalities. [9], Excess abdominal fat, assessed by measurement of waist circumference or waist-to-hip ratio, is independently associated with higher risk for diabetes mellitus and cardiovascular disease [9].

Because of scarce knowledge about the relationship between food consumption patterns and the risk of CAD in Iranian population, in this study we have compared the dietary pattern and nutritional-related heart disease risk factors of established CAD patients with that of matched controls.

## Methods

### Subjects

From July 2004 to march 2006, 108 randomly selected CAD patients (58 male,) and 108 (57 male) matched subjects in the catheterization wards of two main Ahvaz University teaching hospitals were evaluated for their nutritional patterns.

The coronary patients were selected from patients in the catheterization ward of teaching hospitals (with more than 70% stenosis in each of the main coronary vessels or subjects who had been admitted for their myocardial infarction).

We randomly selected sex and age matched individuals who had been referred with atypical chest pain in the same ward and had normal angiography (lower than 40% stenosis in the main coronary vessels) [10,11]. These individuals were chosen as the control group from the same wards rather than selecting healthy controls to have better catchment similarities. Moreover, there were records available from the controls which made the comparisons more precise.

The information regarding medical factors were retrieved from the subjects' medical records, along with lifestyle characteristics, through a confidential and detailed questionnaire administered during physician's interview after the second day of hospitalization for all cases and at the entry for the controls. In order to eliminate recall bias, we tried to retrieve precise information from cases and controls and medical history through the hospital or the personal records.

Current smokers were defined as those who have been smoking at least one cigarette per day. Former smokers were those who had stopped smoking for at least one year, and never smokers were those who had never smoked. Those who had stopped smoking for less than a year were classified as smokers.

Nutritional evaluation was accomplished by a customized Harvard food frequency questionnaire. This questionnaire has previously been validated in national studies [12] and consisted of 41 main food items consumed daily or weekly during the past 6 months. The frequency of consumption was semi-quantified in terms of the number of each food item consumed in a day or week.

Other parameters in all subjects were consisted of arterial blood pressure levels in the right arm (average of 3 measurements having the patient seated and rested), total cholesterol, lipoprotein profile and fasting glucose concentrations. The measurements were collected during the first 72 h of hospitalization. Individuals' past medical histories and their reports assisted us in characterizing the subjects as having hypertension, hypercholesterolemia, or diabetes. In keeping with the long-standing classification criteria used in several population-based studies, patients whose blood pressure, according to their medical records, were greater or equal to 140/90 mmHg or were taking antihypertensive medication were classified as hypertensive [13].

Hypercholesterolemia and hypertriglyceridemia were defined as serum total cholesterol (TC) and triglycerides (TG) levels greater than 200 and 150 mg/dl, respectively, or if hypo-lipidemic treatment was administered. Diabetics were those with fasting blood glucose equal or greater than 126 mg/dl for two times or those who were under diabetic diet or medications. We also measured subjects' anthropometric indices such as height, weight, body mass index (BMI = weight/height^2^), waist/hip ratio (WHR) and body fat percent (BF %) in both patients and controls. The latter was measured using bioelectrical impedance method by Omron BF-302 apparatus, Japan. Obesity was defined as BMI >30 Kg/m^2^. Measurement of the waist circumference as a surrogate for visceral adipose tissue was performed in the horizontal plane above the iliac crest [9].

### Statistical analysis

Estimations regarding effect of each food item were performed by the calculation of odds ratio (OR) and the corresponding 95 percent confidence intervals (95% CI) through multiple conditional logistic regression analysis, adjusting for age, BMI, smoking, history of hypercholesterolemia, hypertension or diabetes. Bivariate regression analysis was conducted for assessing the correlation between two continuous variables. All reported p values were from two-sided tests and compared to a significant level of 5%. SPSS software (version #11.5) was used for statistical analysis.

### Medical Ethics

All participants were informed about the aims of study and they gave their written consents. The study protocol was approved by the Medical Ethics Committee of Ahvaz Jondi-Shapour University.

## Results

The mean age of patients and controls showed no significant difference between the two groups (51.5 ± 9 vs 50.8 ± 9.2 years). Table 1 describes the basic characteristics of both groups.

**Table 1:** Basic characteristics and anthropometric measurements of CAD patients and controls

Variables	Patients	Controls
Age (y)	51.5 ± 9	50.8+9.2

Sex (% male)	(53%) 58	(52%) 57

Education > 12 y	(15%) 17	(35%) 38*

BMI (Kg/m2)	27.64 ± 4.2	25.8 ± 4.3*

WHR (female)	0.95 ± 0.2	0.77 ± 0.06*

WHR (male)	0.94 ± 0.1	0.87 ± 0.07*

BF% (female)	34.69 ± 7	31.67 ± 6*

BF% (male)	28.76 ± 6	22.08 ± 5*

Weight (Kg)	76.13 ± 13	71.89 ± 14*

N1, N2 = 108

* t test, p < 0.05

**Abbreviations: **BMI (body mass index); WHR (waist-to-hip ratio); BF% (body fat percent).

As shown in this table, WHR, BMI, BF and weight of CAD patients were significantly higher than that of their counterpart controls in both sexes.

CAD risk factors of patients and controls are shown in table 2. Prevalence of hypercholesterolemia and hypertriglyceridemia was two times higher in patients (p < 0.001) and almost all patients had high TG and LDL-C levels. Both patients and controls showed higher lipid profiles compared with the established cut-off points. Having diabetes, hypertension, overweight and cigarette smoking habit were significantly higher in CAD patients (p < 0.001).

**Table 2:** Comparison of selected cardiovascular disease risk factors between CAD patients and controls

Variable	Patients (%)	Control (%)	*p *value
TG > 150 mg/dl	99.1	49.5	<0.001

TC > 200 mg/dl	50	25.5	<0.001

LDL-C > 100 mg/dl	99.1	60	<0.001

HDL-C < 35 mg/dl	75.6	25.4	<0.001

FBS > 126 mg/dl*	43.5	10.7	<0.001

BMI > 25 kg/m2	72.9	65.5	<0.002

BP > 140/90 mm Hg	76.9	22.8	<0.001

Current cigarette smoking habit	39.8	7.4	<0.001

(N1, N2 = 108)

**Abbreviations: **TG (triglycerides); TC (total cholesterol); LDL-C (low density lipoprotein-cholesterol); HDL-C (high density lipoprotein-cholesterol); FBS (fasting blood sugar); BMI (body mass index); BP (blood pressure levels).

*FBS concentrations were measured once.

There was no significant association between weekly consumption of red meat, eggs, chicken and daily consumption of fruits and vegetables with the risk of CAD (table 3).

**Table 3:** Comparison of dietary patterns of CAD patients and healthy controls based on food frequency questionnaires

Food groups	Consumption frequency	Patients	Controls	OR(CI 95%)	*p *value
Red meats	>2 times/w	(30) 27.8	(26) 25.7	0.9(0.48-1.66)	0.74

Chicken	>2 times/w	(82) 76.4	(90) 84.1	0.62(0.31-1.22)	0.16

Milk	>1 cup/d	(49) 45.5	(39) 36.4	1.44 (0.83-2.5)	0.18

Full-fat Yoghurt	>1 cup/d	(78) 72.2	(53) 52.5	2.35 (1.32-4.18)	0.003

Fish	>2 times/w	(31) 28.7	(45) 42.1	0.55 (0.31-0.97)	0.04

Fresh Fruits	>1 time/d	(71) 65.7	(71) 66.4	0.97 (0.55-1.71)	0.92

Fresh Vegetables	>1 time/d	(38) 35.5	(46) 43	0.73 (0.42-1.26)	0.26

Eggs	>2 times/w	(32) 29.6	(29) 27.1	0.88 (0.48-1.59)	0.68

Tea (Black)	>3 cups/d	(79) 73.1	(97) 89.9	0.3 (0.15-0.65)	0.002

Hydrogenated fats	Habitual daily usage	(65) 60.2	(45) 41.7	2.13 (1.23-3.64)	0.006

Vegetable oils	Habitual daily usage	(25) 23.1	(60) 56.5	0.23 (0.12-0.42)	0.001

Salads	1 time/d	(37) 34.3	(33) 30.8	0.62(0.31-1.22)	0.59

(N1, N2 = 108)

Figures in parentheses denote number of subjects in each group.

OR (CI 95%) for consuming fish, tea and vegetable oils were 0.55(0.31-0.91), 0.3(0.15-0.65), 0.23(0.13-0.42), respectively. On the other hand, consumption of hydrogenated fats and full-fat yoghurt was associated with higher risk (OR = 2.12 (1.23-3.64) and 2.35 (1.32-4.18), respectively.

In terms of dietary patterns, no differences were found between the two sexes.

## Discussion

The present study revealed that daily consumption of vegetable oils, tea and fish was significantly associated with lower risk of coronary events, even after controlling for potential confounding risk factors (Table 2)

It was also shown that consumption of hydrogenated fats and full-fat yoghurt is associated with significantly higher risk of coronary artery events.

Hypertriglyceridemia and high LDL-C levels were prevalent in almost all CHD patients (99 percent) whilst half of the controls showed hypertriglyceridemia and one-fifth had high LDL-C concentrations. The former might represent significant independent impact of TG levels on CAD.

It has been reported that consumption of fruits and vegetables three or more servings per day versus less than once per day is associated with a 27 percent reduction in cardiovascular disease risk [14]. This finding is also consistent with the results from other studies in which there was a graded risk reduction associated with higher intakes of fruits and vegetables [15-17]. Data suggest that dietary intake of green leafy vegetables and foods rich in carotenoids and vitamin C particularly contributes to this relationship. In most of above mentioned researches, consumption of more than one time vegetables and fruits were compared with those who did not eat vegetables and fruits in a week. However, in present study, daily consumption of vegetables and fruits were compared with those who consumed once a day and there was no significant difference between the groups. At present, less is known about the direct association between fruits and vegetable intake and risk of acute coronary syndrome. The largest long-term randomized dietary intervention trial to date, the Women's Health Initiative (WHI) Randomized Controlled Dietary Modification Trial, [18] was designed to test the hypothesis that a diet low in fat and high in fruit, vegetables and grains would lead to a reduction of cardiovascular disease events. Nearly 50,000 postmenopausal women were randomized to an intervention group receiving regularly scheduled individualized dietary consultations or to a comparison group receiving diet-related education materials only. Despite achieving a reduction in total fat intake between the intervention and control groups (28.8 percent versus 37 percent of calories (*p *< 0.001), respectively), no significant effects of the intervention on CHD, stroke, or CVD were observed during the 8-year follow-up [18].

Despite our attempt to control for several known confounding factors, we could not find any inverse association between fruits and vegetable intake and coronary risk and this could, at least partially, be explained by other factors associated with heart-healthy behaviors. Moreover, in retrospective case-control studies two main sources of systematic errors may exist, the selection and the recall bias. In order to eliminate selection bias we set objective criteria both for patients and controls. However, insignificant misclassification may exist, since a small percentage of asymptomatic coronary patients may be wrongly assigned to controls, even they were evaluated by a cardiologist. Moreover, in case-control studies it is usually observed that patients who had a recent adverse event are more likely to place greater emphasis on several factors related to the disease than the control group (recall bias). To reduce this type of bias and analyze precise information, we obtained accurate information from the patients as well as from their relatives or their accompanying persons. Concerning the medical information, we avoided recall bias by obtaining detailed data from subjects' medical records. However, the coronary patients who died at the entry or the day after were not included into the study. This bias could influence our results.

In our study, consumption of habitual hydrogenated fats and full-fat yoghurts (fat content more than 2.5%) increased the risk of CAD. These foods are the major sources of saturated fatty acids (SAFA). SAFA intake is the principal determinant of TC and CAD and substitution of 1% carbohydrate calories by SAFA increases TC by 1.5 mg/dL [19]. Vegetables oil (olive and canola oils) decrease the risk of CAD. Both olive and Canola oils are high in mono unsaturated fatty acid (MUFA) [20]. Substitution of 1% carbohydrate calories by MUFA lowers TC by 0.5 mg/dL [19]. Replacing SAFA with MUFA may be more effective in preventing CAD than reducing overall fat intake. A higher intake of MUFA could particularly be beneficial among Iranians, because it is very effective in lowering LDL without raising TG or lowering HDL [21].

Mozaffarian and his colleagues [22] showed that partially hydrogenated oils are extensively being used for cooking in Iranian homes with average per-person intake of 14 g/1000 kcal. Trans fatty acids (TFAs) accounted for 33% of fatty acids in these products, or 4.2% of all calories consumed (12.3 g/day). On the basis of TC:HDL-cholesterol effects alone, 9% of CHD events would be prevented by replacement of TFA in Iranian homes with cis-unsaturated fats (8% by replacement with saturated fats). On the basis of relationships of TFA intake with CHD incidence in prospective studies, 39% of CHD events would be prevented by replacement of TFA with cis-unsaturated fats (31% by replacement with saturated fats). However, these population-attributable risks may be overestimates owing to competing risks and because not all the fat used for cooking might actually be consumed. If actual TFAs consumption were only half as large, the estimated proportion of CHD events prevented by TFA elimination would be 5% on the basis of TC:HDL cholesterol effects and replacement with cis-unsaturated (4% for replacement with saturated fats), and 22% on the basis of prospective studies and replacement with cis-unsaturated fats (17% for replacement with saturated fats). These estimates do not include possible additional benefits derived from replacing TFAs with vegetable oils containing n-3 fatty acids [22].

Although several studies in the past have attempted to assess the role of drinking tea on cardiovascular disease incidence, the information remains inconsistent [23,24].

Discussion on possible mechanisms responsible for the effect of tea consumption on the coronary risk is beyond the scope of this paper but some investigator suggest that tea had beneficial effect on cardiovascular system by improving endothelial function [23,24].

A number of studies have reported that consumption of fish, especially species with a high content of omega-3 fatty acids, confers protection from ischemic heart disease and that this relationship is particularly strong for CAD mortality and sudden cardiac death [25]. A recent analysis of the epidemiological data concluded that intake of small quantities of fish is associated with a 17 percent reduction in CHD mortality risk and a 27 percent reduction in risk for nonfatal myocardial infarction, whereas each additional serving per week is associated with a further reduction of 3.9 percent in CHD mortality but no further reduction in risk for myocardial infarction [25]. Although fish has a number of important nutritive qualities, their major cardiovascular benefit likely derives from their content of the omega-3 fatty acids, eicosapentaenoic acid (EPA) and docosahexanoic acid (DHA) [26]. Increased plasma levels of these fatty acids predicted a considerable reduction in sudden cardiac death,[26] a result consistent with that of a report indicating that intake of 5.5 gm/month of EPA plus DHA (equivalent to one portion of fatty fish per week) was associated with a 50 percent lower incidence of primary cardiac arrest compared with individuals consuming no fish [27]. This effect appears to be related to enrichment of membrane phospholipids with omega-3 fatty acids and a resulting reduction in risk for abnormal cardiac electrical conductivity [27] Other properties of these fatty acids that may benefit risk for CAD include anti-platelet aggregation and anti-inflammatory effects, as well as reduction in plasma triglycerides at higher doses [28]. On the basis of these studies, as well as results of intervention trials with omega-3 fatty acids, the AHA has recommended consumption of two portions of fish per week, particularly those fish rich in omega-3 fatty acids.

A more recent work on dietary pattern of 468 middle-age women in Isfahan, Iran revealed that based on food frequency questionnaires, Iranian dietary pattern is significantly associated with a greater risk of dyslipidemia (OR = 1.73) or at least one CVD risk factor (OR = 1.89) and the authors concluded that the dietary patterns may explain the higher prevalence of some cardiovascular risk factors in the region [12].

At present, we know that more than 39 percent of leading causes of death in Iran is attributed to circulatory system disease [29] and our community like similar populations is facing a rapid transition toward chronic diseases especially obesity [30] which could lead to higher prevalence of metabolic syndrome in near future. Hence, it is clear that we need more advanced researches with higher number of subjects in larger epidemiologic settings to demonstrate the relationships between nutritional practices and cardiac diseases in the region.

As a limitation of this study, we propose that there might be a possible bias in random allocation of the matched controls. However, we tried to match the groups in more applicable ways, as described.

Finally, according to the priority of cardiovascular diseases in the region in one hand, and limited findings about diet related practices leading to the risk of CAD in Iran and other Persian Gulf countries on the other hand, present findings can be regarded as one step towards finding better nutritional solutions to control heart diseases. We also need to define specific local cut-off points of CAD to detect our patients with more applied criteria.

## Conclusion

This study revealed that daily consumption of vegetable oils, tea and fish is significantly associated with lower risk of coronary events. On the other hand, it was indicated that consumption of hydrogenated fats and full-fat yoghurt is associated with significantly higher risk of coronary artery events.

Our CAD patients significantly have higher body fat percent, body mass index and waist-to-hip ratio compared with their matched controls.

The results also showed that in terms of lipid profile cut off-points, both patients and controls have high levels of riskfactors which needs more attention regarding cardiac disease outcomes.

## Competing interests

The authors declare that they have no competing interests.

## Authors' contributions

RA made the preliminary conception and study design and supervised the research. MN and SR did the data collection. NA was the clinical supervision as a cardiologist and MHH carried out the statistical analysis. All authors read and approved the final manuscript.
